# Biological and Enzymatic Characterization of Proteases from Crude Venom of the Ant *Odontomachus bauri*

**DOI:** 10.3390/toxins7124869

**Published:** 2015-11-30

**Authors:** Mariana Ferreira Silva, Caroline Martins Mota, Vanessa dos Santos Miranda, Amanda de Oliveira Cunha, Maraísa Cristina Silva, Karinne Spirandelli Carvalho Naves, Fábio de Oliveira, Deise Aparecida de Oliveira Silva, Tiago Wilson Patriarca Mineo, Fernanda Maria Santiago

**Affiliations:** 1 Institute of Biomedical Sciences, Laboratory of Immunoparasitology “Dr. Mario Endsfeldz Camargo”, Federal University of Uberlândia, Av. Pará 1720, Uberlândia 38400-902, Brazil; marianaa_fs@hotmail.com (M.F.S.); carolinemartinsm@yahoo.com.br (C.M.M.); vanessa.smiranda@hotmail.com (V.S.M.); amanda.olicunha@hotmail.com (A.O.C.); maraisa2003@yahoo.com.br (M.C.S.); daosilva@yahoo.com.br (D.A.O.S.); tiagomineo@icbim.ufu.br (T.W.P.M.); 2 Institute of Biomedical Sciences, Laboratory of Clinical Bacteriology, Federal University of Uberlândia, Av. Pará 1720, Uberlândia 38400-902, Brazil; kscnaves@icbim.ufu.br; 3 Institute of Biomedical Sciences, Laboratory of Biophysics, Federal University of Uberlândia, Av. Pará 1720, Uberlândia 38400-902, Brazil; foliveira@umuarama.ufu.br; 4 National Institute in Science and Technology in Nanobiopharmaceutics (NanoBiofar), Belo Horizonte-MG 31270-901, Brazil

**Keywords:** *Odontomachus bauri*, crude venom, proteases, *Toxoplasma gondii*

## Abstract

Hymenoptera venoms constitute an interesting source of natural toxins that may lead to the development of novel therapeutic agents. The present study investigated the enzymatic and biological characteristics of the crude venom of the ant *Odontomachus bauri*. Its crude venom presents several protein bands, with higher staining for six proteins with gelatinolytic activity (17, 20, 26, 29, 43 and 48 kDa). The crude venom showed high proteolytic activity on azocasein at optimal pH 8.0 and 37 °C. In the presence of protease inhibitors as aprotinin, leupeptin and EDTA, the azocaseinolytic activity was reduced by 45%, 29% and 9%, respectively, suggesting that the enzymes present in the crude venom belong to the three classes of proteases, with the serine proteases in greater intensity. The crude venom degraded the fibrinogen α-chain faster than the β-chain, while the fibrinogen γ-chain remained unchanged. In biological assays, *O. bauri* venom showed hemolytic and coagulant activity *in vitro*, and defibrinating activity *in vivo*. In addition, the venom showed antimicrobial activity against *Staphylococcus aureus* and *Escherichia coli* as well as antiparasitic activity on *Toxoplasma gondii* infection *in vitro*. In that sense, this study sheds perspectives for pharmacological applications of *O. bauri* venom enzymes.

## 1. Introduction

Ants of the genus *Odontomachus* are widely distributed in tropical and warm countries, being especially abundant in the neotropics [1,2]. Ants of the species *O. bauri* usually build their nests in the ground, protecting them from direct sunlight and choosing the place to build them far from environmental disturbance. When nests are disturbed, these ants attack their aggressors and their bites cause immediate acute pain and a burning sensation [3].

Also known as trap jaw ants, their movements are extremely fast and produce remarkably predatory attacks [4,5]. During predatory strikes, *O. bauri* mandibles close at a speed ranging from 35 to 64 m/s, far surpassing the speeds of other ballistic predatory appendages already documented in the animal kingdom [5], including the discharge of the cnidarian nematocyst [6].

In addition, these ants have the ability to disable prey because their mandibles were evolutionarily adapted for locomotion. *O. bauri* specimens use their claws to perform some jumps, which have the assumed forms of “bouncer defense” [7].

The venom of *O. bauri* and other insects of the order Hymenoptera, is produced in venom glands (structure located in the last segment of the body), wich are formed from modified accessory glands of the female reproductive system [8,9,10]. Ant species of the genus *Odontomachus* are particularly aggressive and their venoms have high toxic activity [3]. These ants produce various chemicals that are used for attack, defense and communication through volatile components in prey capture, protect the nest from predators and prevent the development of diseases in their colonies [11]. It is known that the venom comprises organic molecules such as proteins, lipids, vasoactive amines and a wide variety of different enzymes, such as phospholipases and hyaluronidases [12,13,14,15,16,17]. These substances are responsible for the toxicity of this venom and several of these components have pharmacological and therapeutic properties [18]. The mapping recent of the *Tetramorium bicarinatum* ant crude venom demonstrated the presence of different proteins, including toxin (11%) and non-toxin (3%) class proteins. With regard to toxin class, the authors observed a high diversification with the major part consistent with the classical hymenopteran venom protein signature represented by venom allergen (33.3%), followed by a diverse toxin-expression profile including several distinct isoforms of phospholipase A1 and A2, venom serine protease, hyaluronidase, protease inhibitor and secapin [19].

Considering the essential role of insect proteases for survival and death of living organisms, along with the increasing importance as potential therapeutic targets, the aim of the present work was to investigate the biological and enzymatic characteristics of proteases present in the crude venom of the ant *O. bauri*.

## 2. Results

### 2.1. Electrophoretic Profile

**Figure 1:**
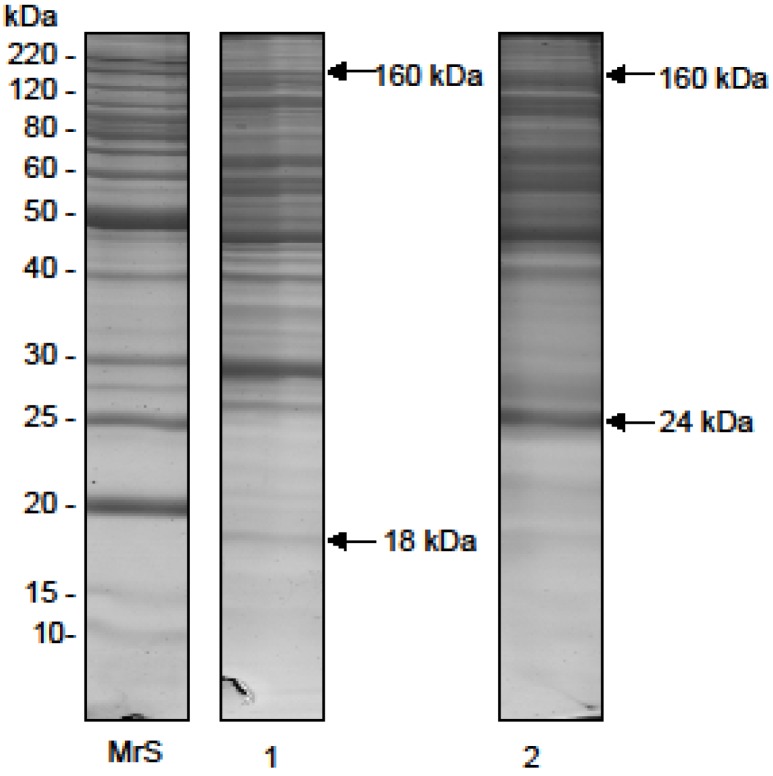
Electrophoretic profile of the *O. bauri* venom*.* Silver stained SDS-polyacrylamide gel at 14%. Venom samples of *O. bauri* (15 µg) were analyzed in non-reducing and reducing (2-mercaptoethanol) conditions. MrS: molecular size markers; lane 1, crude venom of *O. bauri* in non-reducing conditions; lane 2, crude venom of *O. bauri* in reducing conditions.

The *O. bauri* crude venom samples from several extractions had a mean protein concentration of 715.0 µg/mL. The SDS-PAGE profile of *O. bauri* crude venom showed several peptide components, with relative molecular masses (*Mr*) ranging from 18 to 160 kDa when analyzed under nonreducing conditions, with more intense staining for bands above 29 kDa (Figure 1, lane 1). Under reducing conditions, the electrophoretic profile was changed, showing a wider *Mr* range, from 24 to 160 kDa, (Figure 1, lane 2).

### 2.2. Enzymatic Activities

#### 2.2.1. Azocaseinolytic Activity

**Figure 2:**
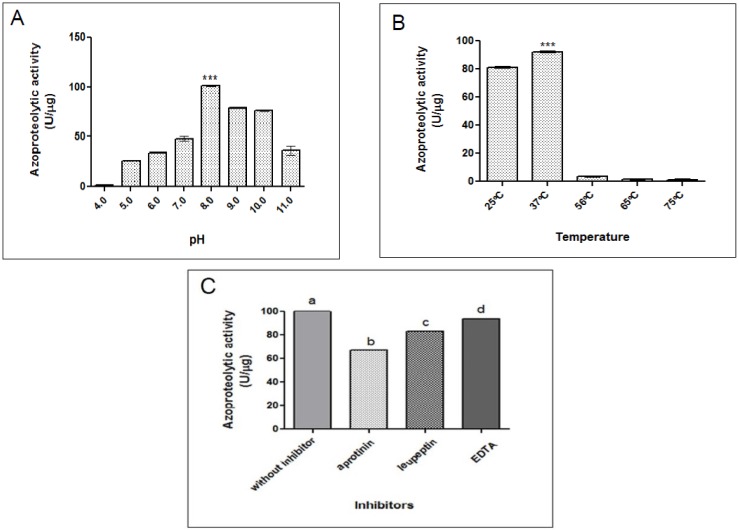
Influence of pH, temperature and inhibitors on the proteolytic activity of the *O. bauri* crude venom on azocasein. (**A**) The crude extract (1 µg) was preincubated at various ranges of pH; (**B**) different temperature or (**C**) with different inhibitors (5 mM) for 30 min and added to azocasein (1 mg/mL) for 60 min at 37 °C. The azocaseinolytic activity was assayed at 405 nm and expressed in U/µg. Results are reported as mean ± standard deviation. *** Statistically significant differences in comparison to other ranges of pH or temperature (*p* < 0.0001). In (**C**), different letters indicate statistically significant differences among the inhibitors (*p* < 0.05) (ANOVA and Bonferroni multiple comparison post-test).

The proteolytic activity of *O. bauri* crude venom on azocasein was determined as 102 U/µg. When evaluating the effect at various pH the venom presented higher and optimal activity in pH 8.0, with a significant loss in acidic (4.0; 5.0 and 6.0) and basic (11.0) pH (Figure 2A). The effect of temperature in the proteolytic activity showed high activities between 25 °C and 37 °C, with optimal activity at 37 °C and significant reduction at higher temperatures (Figure 2B). In this way, the following experiments were performed at 37 °C for one hour.

Concerning the effect of inhibitors, the proteolytic activity was significantly reduced after pre-incubation with aprotinin, leupeptin and EDTA. However, aprotinin allowed the highest reduction of the activity (45%) when compared to other inhibitors as leupeptin (29%) and EDTA (9%) (Figure 2C). In contrast, the effect of ions as Ca^2+^, Mg^2+^, Zn^2+^ and Cu^2+^ did not show any change of the proteolytic activity (data not shown).

#### 2.2.2. Gelatin Zymography

**Figure 3:**
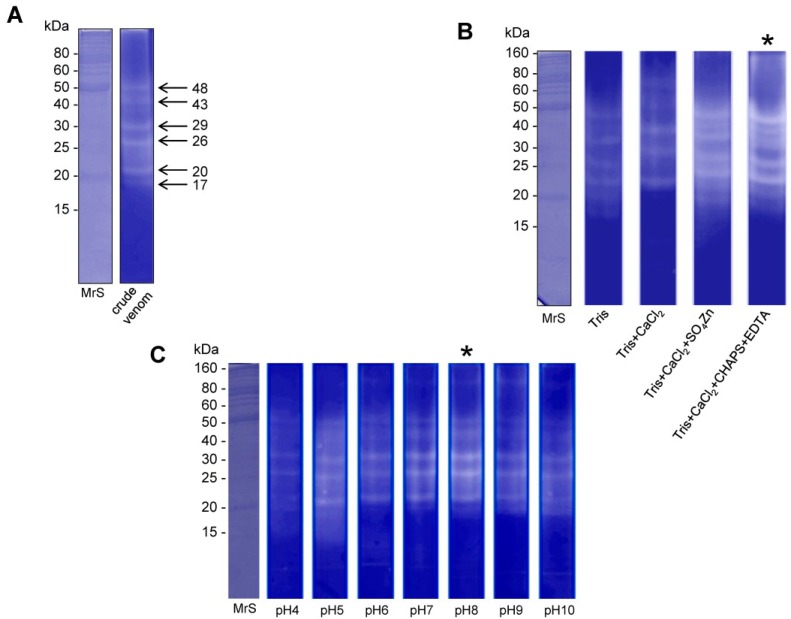
Acrylamide-gelatin gel zymography of the *O. bauri* venom. (**A**) Crude venom samples were analyzed in non-reducing conditions. MrS: molecular size markers; (**B**) Effect of different buffers (50 mM Tris-HCl; 50 mM Tris-HCl and 10 mM CaCl_2_; 50 mM Tris-HCl, 1 mM CaCl_2_ and 1 mM SO_4_Zn; 50 mM Tris-HCl, 150 mM NaCl, 10 mM CaCl_2_, 0.002%CHAPS and 10 mM EDTA) on the gelatin proteolysis activity of the *O. bauri* venom; (**C**) Effect of different ranges of pH (4 to 10) on the gelatin proteolysis activity of the *O. bauri* venom. (*) optimal buffer and pH.

The Zymogram method was used to determine the nature and the molecular weight of the gelatinolytic enzyme present in the venom of *O. bauri*. Figure 3 shows that the crude venom presented six proteins having gelatinolytic activity, with apparent molecular masses of 17, 20, 26, 29, 43 and 48 kDa (Figure 3A). When the effect of different buffers and pH (4.0–10.0) in the gelatinolytic activity was evaluated, we observed increased renaturation of proteases with buffer containing CaCl_2_ and NaCl in the presence of the chemicals CHAPS and EDTA (Figure 3B) and with optimal pH of 8.0 (Figure 3C).

**Figure 4:**
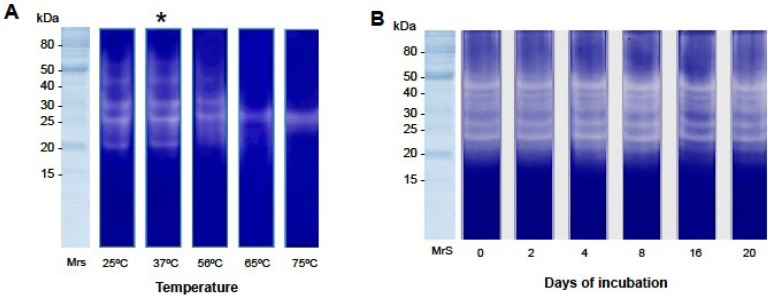
The temperature effect on the gelatin proteolysis activity of the *O. bauri* venom and enzyme stability. (**A**) Temperature-dependent gelatin zymography. Crude venom samples (5 µg) were incubated at different temperatures for 30 min. MrS: molecular size markers; (*) optimal temperature for enzymatic activity; (**B**) Enzyme stability. Crude venom samples were incubated at 4 °C in intervals from 2 to 20 days and applied to the gel of gelatinase activity.

Temperature was also critical for gelatin proteolysis induced by the *O. bauri* crude venom, as we demonstrate that its activity was maintained between 25 °C and 37 °C after 30 min of reaction. However, this activity was impaired when the temperature increased above 56 °C (Figure 4A). Enzyme stability was also evaluated, and showed that the *O. bauri* venom was constant until the 20th day of incubation at 4 °C (Figure 4B).

#### 2.2.3. Fibrinogenolytic Activity

**Figure 5:**
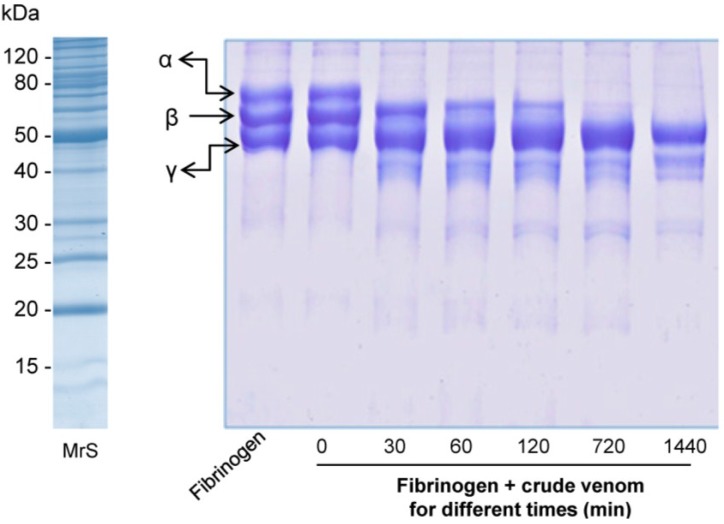
Proteolysis of bovine fibrinogen by the *O. bauri* crude venom. Fibrinogen was incubated or not with 5 µg of the crude venom of *O. bauri* at 37 °C for 0, 30, 60, 120, 720 (12 h) and 1440 (24 h) min and then analyzed on SDS-PAGE (14%). MrS: molecular size markers; bovine fibrinogen chains (α, β, and γ) are shown on the left.

Crude venom of *O. bauri* showed a time-dependent fibrinogenolytic activity. The enzymes completely degraded bovine fibrinogen α-chain at a concentration of 5 μg and with 30 min of incubation. However, degradation of fibrinogen β-chain was observed with longer incubation time (720 min) and on the other hand, the enzymes did not showed any activity over fibrinogen γ-chain (Figure 5).

### 2.3. Biological Activities

#### 2.3.1. Hemolytic Activity

**Figure 6:**
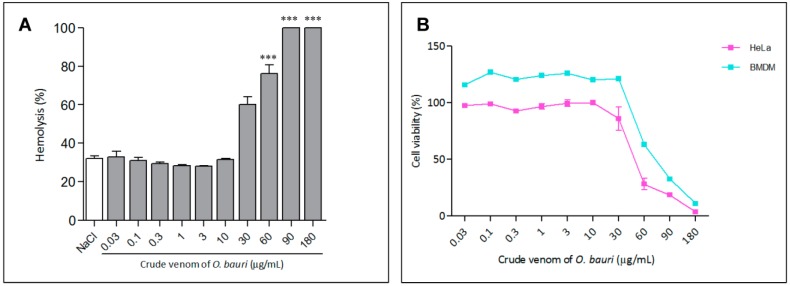
Hemolytic activity and *in vitro* cytotoxicity. (**A**) Red blood cells were incubated with different concentrations (0.06 to 32 µg/mL) of the crude venom or 1% (*v*/*v*) Triton X-100 (total lysis) or in the presence of 0.9% NaCl (spontaneous lysis). Absorbance was measured at 540 nm and results (mean ± SD) are reported as percentage of hemolysis in relation to total lysis. *** *p* < 0.0001 in relation to NaCl control (ANOVA and Dunett post-test); (**B**) HeLa cells and murine bone-marrow-derived macrophages were separately cultured in 96-well plates in the absence (control) or presence of different concentrations of the *O. bauri* crude venom (0.03, 0.1, 0.3, 1, 3, 10, 30, 60, 90 and 180 µg/mL) for 24 h. The results were expressed as the percentage of viable cells in relation to the control.

The hemolytic activity of *O. bauri* crude venom was verified in different concentrations, reaching a maximal hemolytic activity (around 100% lysis) from the concentration from 60 to 180 µg/mL (*p* < 0.01) (Figure 6A).

#### 2.3.2. Cell Viability Assay

Viability of HeLa cells and murine bone marrow-derived macrophages (BMDM) in the presence of different concentrations of *O. bauri* crude venom (Figure 6B) was above 63% and 85%, respectively, even when the highest concentrations (30 and 60 µg/mL) were used.

#### 2.3.3. Hemorrhagic and Coagulant Activities

The crude extract of *O. bauri* was also evaluated for hemorrhagic and coagulant activities. There was no formation of minimum hemorrhagic lesion (above 10 mm of diameter) in Swiss mice inoculated intradermally with the crude venom, even using high concentration (50 µg). However, the *O. bauri* venom was able to coagulate bovine plasma in about 15 sec when compared to the positive control containing 0.2 M CaCl_2_ (coagulation in about 2 min) (data not shown).

#### 2.3.4. Defibrinating Activity

Crude venom of *O. bauri* caused defibrinogenation when administered intraperitoneally to mice, making the plasma uncoagulable. Animals treated with the venom promoted blood clotting after 4.3 min while the control animals had average clotting time of 1.6 min (*p* < 0.01) (data not shown).

#### 2.3.5. Antimicrobial Activity

The antimicrobial activity of the *O. bauri* crude venom was also examined and the results were measured by the zones of bacterial growth inhibition around each of the disks, comparing with positive controls. *O. bauri* crude venom presented antimicrobial activity against both Gram-negative (*E. coli*) and Gram-positive (*S. aureus*) bacteria in the concentration of 15 μg/disk, with inhibition of bacterial growth in 62.5% and 72.7%, respectively, when compared to positive controls (Table 1).

**Table 1:** Antimicrobial activities of the *Odontomachus bauri* crude venom by using the agar diffusion technique.

*O. bauri* Venom Concentration (μg)	Zones of Growth Inhibition, in mm (% Inhibition)	
	*Escherichia coli*	*Staphylococcus aureus*
15	15 (62.5)	16 (72.7)
10	12 (50.0)	14 (63.6)
5	11 (45.8)	11 (50.0)
2.5	0	0
1.25	0	0
0.6	0	0
0.3	0	0
Positive control *	24	22

* Oxacylin (*S. aureus*) and Ampicylin (*E. coli*).

#### 2.3.6. Antiparasitic Activity

**Figure 7:**
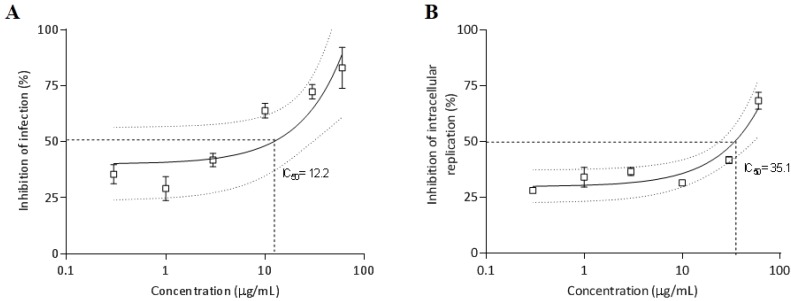
Effect of pretreatment of *T. gondii* tachyzoites with *O. bauri* crude venom in different concentrations (0.3, 1, 3, 10, 30 and 60 µg/mL) or with medium alone (control) (**A**) on *T. gondii* infection and (**B**) intracellular replication in HeLa cells. Results are expressed as mean (box) and standard deviation of the percentages of inhibition of infection and intracellular replication related to controls. Dotted lines show the inhibitory concentration of 50% (IC_50_).

Effect of the *O. bauri* crude venom on *T. gondii* infection and replication in HeLa cells was verified and shown in Figure 7. The pretreatment of *T. gondii* tachyzoites with *O. bauri* venom before infection of HeLa cells showed a dose-response inhibitory curve that reached up to 83% of inhibition and showed an IC_50_ of 12.2 µg/mL for the infection index (Figure 7A). Concerning the inhibition of intracellular parasite replication, the pretreatment of *T. gondii* tachyzoites with *O. bauri* before infection of HeLa cells showed a dose-dependent inhibition, reaching rates of 68% and IC_50_ of 35.1 µg/mL (Figure 7B).

## 3. Discussion

Hymenoptera venoms constitute a number of pharmacologically active biomolecules, from which the most common components are low molecular weight proteins recognized as important allergens and resulting in an IgE-mediated reaction [20,21,22]. The discovery of such natural toxins may lead to the identification of model compounds for the development of novel therapeutic agents [23]. In that sense, we evaluated the role of the crude venom of the ant *O. bauri*, concerning their biological and enzymatic characteristics.

First, the electrophoretic profile of the *O. bauri* crude venom revealed several peptide bands between 18 and 160 kDa. Insect venoms contain numerous proteins with or without enzymatic activity, and usually have abundant protein profiles. Previous studies reported that crude venoms of other species of ants, such as *Solenopsis invicta* and *Myrmecia pilosula,* also exhibited an extensive electrophoretic profile with bands ranging from 10 to 232 kDa [10,24,25].

Second, the effect of pH on the proteolytic activity of the crude venom on azocasein substrate was evaluated, showing an optimal activity at pH 8.0. Similar results were found in other species of Hymenoptera. Whitwort *et al.* [26] found an optimum pH of 8.0 for a protease isolated from the larvae of ant *Solenopsis invicta*, and observed ability of gelatin and azocasein degradation by the enzymes of the venom. The evaluation of the effect of temperature on the gelatin proteolysis activity of the crude venom showed high activities between 25 °C and 37 °C, optimal activity at 37 °C and impaired activity above 56 °C. Qiu *et al.* [27] reported optimum temperature at 30 °C for a serine protease isolated from the venom of bee *Bombus terrestris.* Above this temperature the enzyme activity declined sharply, because high temperatures can cause protein denaturation [28].

The enzymatic activity of the *O. bauri* crude venom in the presence of different protease inhibitors (EDTA, leupeptin, and aprotinin) showed significant reduction under effect of these inhibitors, particularly aprotinin, suggesting that the crude venom presents serine proteases in greater intensity or alternatively, this could also be indicative of potent proteolytic activity of the serine proteases. According to Bouzid *et al.* [29] some proteins/enzymes present in the venom of ant *Tetramorium bicarinatum* are components such as sialidase, prophenoloxidase and serine protease. Snake venom proteolytic enzymes are generally composed by two major groups: serine proteases and metalloproteases. Recent work demonstrated that the proteolytic activity of serine proteases Da-36 of the *Deinagkistrodon acutus* snake venom was strongly reduced by the inhibitor PMSF and moderately affected by benzamidine and aprotinim [30].

The crude venom of *O. bauri* also presented gelatinolytic activity as determined by the zymogram method, showing proteins with apparent molecular masses ranging from 17 to 48 kDa. Assays of the effect of different buffers and pH in the gelatinolytic activity showed increased renaturation of proteases with the use of buffers containing CaCl_2_ and NaCl in the presence of CHAPS and EDTA and optimal pH at 8.0. Previous studies reported that the detergent CHAPS and the ionic strength generated by NaCl can modulate the activity and stability of some proteins [31].

The fibrinogenolytic activity assay showed that the enzymes of the *O. bauri* crude venom were able to degrade the fibrinogen α-chain and β-chain, while the fibrinogen γ-chain remained unchanged, suggesting that these enzymes may be grouped as α and β class fibrinogenases. Similar results demonstrated that serine protease isoenzymes purified of the *Daboia russelii russelii* snake venom preferentially cleaved α-chain of fibrinogen with a lower activity towards fibrinogen β-chain [32].

Concerning the biological activities, the crude venom of *O. bauri* showed cytotoxic effects for HeLa cells and BMDM by MTT assays and maximal hemolytic activity, only when administered in high concentration (60 µg/mL). The ability to cause lyses and hemolysis appears to be physiologically important, suggesting that the enzymes present in the venom interact with cell membranes and cause disorder in their organization, leading to rupture [33]. Venom of some ants of the subfamily Ponerinae such as *Dinoponera grandis*, *Platythyrea. cribinodis*, *araponeractatomma* and *Odontomachus hematodus* exhibit hemolytic activity; however, this activity is low compared to other Hymenoptera venoms, such as those of the social wasps [34,35].

When studying the hemorrhagic activity of the *O. bauri* crude venom, the enzymes were not able to degrade proteins from extracellular matrix of basal endothelial cells and consequently induce hemorrhagic lesions. The absence of hemorrhagic activity was verified in venoms of other ant species, such as *Pogonomyrmex barbatus* and *Paraponera clavata* and wasps, such as *Vespula pensylvanica* and *Polystes flavus* [36]. On the other hand, the crude venom of *O. bauri* showed coagulant activity *in vitro* and defibrinating activity *in vivo*, allowing future studies on thrombolytic diseases. Enzymes with anticoagulant properties have been described for some ant venoms, such as *Pogonomyrmex barbatus*, wasps as *Vespula pensylvanica*, *Polystes flavus* [36] and *Vespa magnifica* [37] as well as snakes like *Bothrops* [38]. Serine proteases generally cause defibrinating activity *in vivo*, as observed in the *Bothrops asper* snake venom, but also are able of promote blood clotting *in vitro* [39].

The *O. bauri* crude venom showed antimicrobial activity against *S. aureus* and *E. coli*, supporting the biological activity of its enzymatic compounds. Recent studies have demonstrated that ant *Myrmecia pilosula* peptides exhibited moderate antimicrobial activity against *Escherichia coli* and *Staphylococcus aureus* [10]. The antimicrobial activity of mastoparans, a family of small peptides identified from the venom of hymenopteroid insects, has been reported [40], due to interaction between the positively and negatively charged microbial membranes. This is the first report of the activity of the *O. bauri* venom against both Gram-positive and Gram-negative bacteria, although the actual antimicrobial mechanism is still unclear.

Finally, the *O. bauri* venom also showed antiparasitic activity on *T. gondii* infection *in vitro*. The pretreatment of *T. gondii* tachyzoites with the venom before infection of HeLa cells was able to control the infection, as demonstrated by dose-dependent inhibition curves and considerably low IC_50_ values. Similar effect was observed concerning the dose-dependent inhibition of parasite intracellular replication. These findings indicate that the *O. bauri* crude venom showed to be effective when tested directly against the parasite, with more reduction in the infection index than the parasite replication. A recent study reported that *Bungarus caeruleus* snake venom (BCV) possessed anti-leishmanial activity against promastigotes and amastigotes of *Leishmania donovani*, with BCV IC_50_ values of 14.5 µg/mL and 11.2 µg/mL, respectively [41].

## 4. Experimental Section

### 4.1. Animals

Male Swiss mice (18–22 g) were kept in the Bioterism Center and Animal Experimentation, Federal University of Uberlândia, MG, Brazil. All procedures were conducted according to guidelines for animal ethics and the study received approval of the Ethics Committee for Animal Experimentation of the institution (protocol number 059/14).

### 4.2. Crude Venom

The ants (*O. bauri*) were collected in Uberlândia city, Minas Gerais state, Brazil, and immediately frozen and stored at −20 °C. The venom gland of *O. bauri* was obtained by removing the sting apparatus with an entomological forceps, grabbing the last segment of the abdomen and detaching it, along with the sting apparatus. The venom samples were extracted from a quantity of 10 ants, solubilized with physiological saline solution (0.9% NaCl, Sigma-Aldrich, St. Louis, MO, USA) and centrifuged at 13,000× *g* for 10 min. Venom protein concentrations were determined by the method of [42], using bovine serum albumin as standard.

### 4.3. Polyacrylamide Gel Electrophoresis (SDS-PAGE)

The gels were prepared using the system of discontinuous buffer described by Laemmli [43]. The stacking gel was prepared with 4% acrylamide-bisacrylamide (Sigma-Aldrich, St. Louis, MO, USA), whereas for the separating gel a concentration of 12% was used. Venom samples (20 µg) were loaded by track in gels. Gels were run under both reducing (with β-mercaptoethanol, Sigma-Aldrich) and non-reducing conditions. Proteins were stained with a solution of Coomassie blue R-250 (Sigma-Aldrich). Molecular size markers (MrS) (BenchaMarckTM Protein Ladder, Invitrogen, Carlsbad, CA, USA) were used in each electrophoretic run.

### 4.4. Enzymatic Activities

#### 4.4.1. Azocaseinolytic Activity

Proteolytic activity of the *O. bauri* venom was determined using azocasein (Sigma-Aldrich) as substrate [44] with modifications. Aliquots of 1 µg of venom were added to a mixture of 500 µL of 50 mM Tris-HCl (Sigma-Aldrich) pH 6.8 and 500 µL of 2% azocasein solution (*w*/*v*). As negative control, 500 µL of saline solution were added to 500 µL of 2% azocasein solution. After 1 h of incubation at 37 °C the reaction was stopped by adding 100 µL of 15% trichloroacetic acid (TCA, Sigma-Aldrich) and the samples were centrifuged at 10,000× *g* for 10 min. One unit of activity was defined as an increase of 0.01 in absorbance units at 405 nm, and the results were expressed as specific activity units (U/mg).

#### 4.4.2. Effect of pH and Temperature on Azocaseinolytic Activity

To study the effect of pH on azocaseinolytic activity, 1 µg of venom was added to 500 µL of 2% azocasein solution buffered with 500 µL of the following buffers at various pH ranges: 0.2 M sodium acetate (Sigma-Aldrich) pH 4.0 and pH 5.0; 0.2 M sodium phosphate (Sigma-Aldrich) pH 6.0; 0.2 M Tris-HCl pH 7.0 and pH 9.0; 0.2 M sodium borate (Sigma-Aldrich) pH 10.0; 0.2 M phosphate sodium (Sigma-Aldrich) pH 11.0.

The effect of temperature on the azoproteolytic activity was verified by preheating for 15 min 1 µg of venom at temperatures ranging from 25 °C to 75 °C, following incubation with 2% azocasein solution. The reactions were stopped by adding 100 µL of 15% TCA, followed by centrifugation and the absorbance read in a spectrophotometer at 405 nm.

#### 4.4.3. Effect of Inhibitors and Ions on Azocaseinolytic Activity

The stability of enzymes of the *O. bauri* venom was evaluated on the basis of its proteolytic activity on azocasein in the presence of different protease inhibitors as aprotinin (serine proteases, Sigma-Aldrich), leupeptin (cysteine proteases, Sigma-Aldrich) and EDTA (metalloproteases, Sigma-Aldrich) and bivalent ions (Ca^2+^, Mg^2+^, Zn^2+^ and Cu^2+^), all reagents at concentration of 5 mM. Aliquots of 1 µg of venom and 5 µL of inhibitors or ions were preincubated for 15 min and then solubilized in 2% azocasein solution. After 1 h of incubation the reaction was stopped and the enzymatic activity determined as above described.

#### 4.4.4. Gelatin Zymography

The technique described by [45], with some modifications, was employed, using gelatin as substrate. Crude venom samples (5 µg) were separated by 12% SDS-PAGE containing 1% of the gelatin substrate (Sigma-Aldrich). Subsequent to the electrophoresis, the gel was washed twice for 30 min at room temperature in 2.5% Triton X-100 (Sigma-Aldrich) to remove the SDS and incubated at 37 °C for 18 h in one of the following buffers: 0.05 M sodium citrate pH 4.0, pH 5.0 and pH 6.0; 0.05 M Tris-HCl pH 7.0, pH 8.0, pH 9.0 and pH 10.0; and in the presence of ions and other chemicals as 50 mM Tris-HCl pH 8.0; 50 mM Tris-HCl and 10 mM CaCl_2_ (Sigma-Aldrich) pH 8.0; 50 mM Tris-HCl, 150 mM NaCl, 10 mM CaCl_2_, 0.002% CHAPS (Sigma-Aldrich) and 10 mM EDTA pH 8.0; and 50 mM Tris-HCl, 1 mM CaCl_2_ and 1 mM ZnSO_4_ (Sigma-Aldrich) pH 8.0. The gels were stained with R-250 Coomassie blue and gelatin proteolysis activity detected as colorless bands in the otherwise blue gel.

#### 4.4.5. Temperature Dependent Gelatinolytic Activity

The thermal effect on the gelatin proteolysis activity of the *O. bauri* venom was investigated at temperatures from 25 °C to 75 °C. First, aliquots (20 µg) were incubated at different temperatures (25 °C, 37 °C, 56 °C, 65 °C and 75 °C) for 30 min and applied to the gel of gelatinase activity. After electrophoresis, the gel was incubated with 50 mM Tris-HCl, 150 mM NaCl, 10 mM CaCl_2_, 0.002% CHAPS and 10 mM EDTA (pH 8.0) for 18 h and stained with Coomassie blue.

#### 4.4.6. Enzyme Stability

To analyze the enzyme stability, 200 µL of the *O. bauri* venom (stock solution at 500 µg/mL) was incubated at 4 °C in intervals from 2 to 20 days. At each day of incubation, aliquots of 10 µL (5 µg) were removed and applied to the gel of gelatinase activity as above described.

#### 4.4.7. Fibrinogenolytic Activity

The fibrinogenolytic activity of the *O. bauri* venom was determined in SDS-PAGE according to the methodology of [46], with modifications. Briefly, 25 µL of bovine fibrinogen (stock solution at 3 mg/mL, Sigma-Aldrich) were incubated with 5 µg of the venom at 37 °C. At different time intervals (30, 60, 120, 720 and 1440 min), aliquots were collected and the reaction was stopped by adding SDS sample buffer. The hydrolysis profile was followed by SDS-PAGE at 12% gel [43].

### 4.5. Biological Activities

#### 4.5.1. Hemolytic Activity

Red blood cells of Swiss mice were used to evaluate the hemolytic activity of the crude venom of *O. bauri* according to [47] with modifications. After collected the red blood cells were washed twice with 0.9% NaCl (*w*/*v*) and 0.5% erythrocytes (*v*/*v*) were incubated at 37 °C in the presence of venom (32 to 0.06 µg) for 1 h. Samples were then centrifuged (450× *g* for 5 min), and the absorbance of the supernatants was measured at 540 nm. The absorbance measured from lysed red blood cells in presence of 1% (*v*/*v*) Triton X-100 was considered as 100%.

#### 4.5.2. Cell Viability Assay

Cytotoxicity of *O. bauri* crude venom was assessed by determining cellular viability using MTT assay as previously described [48]. HeLa cells and BMDM from Swiss mice were cultured separately in 96-well plates (1 × 10^5^ cells/well) in triplicate, in the presence of *O. bauri* crude venom in different concentrations (0.03, 0.1, 0.3, 1, 3, 10, 30, 60, 90 and 180 µg/mL). As controls, cells were incubated with complete RPMI medium alone. After 24 h of incubation at 37 °C and 5% CO_2_, cells were washed and pulsed with 10 µL of thiazolyl blue at 5 mg/mL in 90 µL of complete RPMI medium 4 h prior to the end of the culture. Formazan particles were solubilized in 10% sodium dodecyl sulfate (SDS) and 50% *N*,*N*-dimethyl formamide (Sigma-Aldrich). The optical density was read after 30 min at 570 nm in a plate reader (Titertek Multiskan Plus, Flow Laboratories, McLean, VA, USA). Results were expressed as percentage of cell viability in relation to controls.

#### 4.5.3. Hemorrhagic Activity

The hemorrhagic activity was assessed according to [36]. Samples containing 50 μg of the crude venom of *O. bauri* were prepared in 0.9% NaCl and injected intradermally into the dorsal skin of Swiss mice, and saline solution alone was used as negative control. Three hours after the injection, the animals were sacrificed by cervical dislocation and the dorsal skin was removed. The minimum hemorrhagic dose (MHD) was defined as the amount of protein that induced a hemorrhagic lesion of 10 mm of diameter, as calculated using the perpendicular major diameters of the hemorrhagic spot.

#### 4.5.4. Coagulant Activity

The coagulant activity of venom was assessed on citrated bovine plasma as described by Denson *et al.* [49], with modifications. Samples of 20 µg of the crude venom of *O. bauri* were added to aliquots of 200 µL of bovine plasma and incubated at 37 °C. The activity was characterized by the immediate appearance of fibrin network compared with the clotting time of the control containing 0.2 M CaCl_2_.

#### 4.5.5. Defibrinating Activity

The defibrinating activity of venom was tested by the method of [50], with modifications. The activity was assessed by intraperitoneal injection of 2 μg/g body weight of mice of the *O. bauri* crude venom in 100 μL of saline solution into male Swiss mice (18–22 g), using three mice per group; control animals received 200 μL of saline solution. After one hour, the animals were anesthetized and submitted to cardiac puncture. Blood was placed in tubes and kept at 25–30 °C until clotting occurred. The minimum defibrinating dose (MDD) was defined as the amount of venom able to prevent coagulation.

#### 4.5.6. Antimicrobial activity

The antimicrobial activity of the *O. bauri* venom was performed by the disk diffusion susceptibility method according to Yagmur *et al.* [51], with modifications, by applying a bacterial inoculum of approximately 2 × 10^8^ CFU/mL to the surface of a large (150 mm diameter) Mueller-Hinton agar plate. Bacteria specimens tested included a Gram-positive, *Staphylococcus aureus* (ATCC 25923) and a Gram-negative, *Escherichia coli* (ATCC 25922). Paper filter disks (0.5 mm diameter) were prepared with crude venom of *O. bauri* in the following concentrations: 15, 10, 5, 2.5, 1.25, 0.6 and 0.3 μg per disk unit; and placed on the inoculated agar surface. Commercially-prepared disks were used as positive control for *S. aureus* (Oxacillin; 1 μg, Laborclin, Pinhais, Brazil) and *E. coli* (Ampicillin; 10 μg, Laborclin). Sterile water disks as negative control were applied to both bacteria. Plates were incubated for 16–24 h at 35 °C prior to determination of results by measuring the zones of growth inhibition around each of the disks.

#### 4.5.7. Antiparasitic Activity

The antiparasitic activity of the *O. bauri* venom was verified on *in vitro* *T. gondii* infection following the protocols of de Oliveira *et al.* [52]. HeLa cells were cultured on 13-mm round glass coverslips into 24-well plates (1 × 10^5^ cells/well/200 µL) for 24 h at 37 °C and 5% CO_2_. *T. gondii* tachyzoites (RH strain) were obtained from previously infected HeLa cells, washed in RPMI medium and pretreated for 1 h at 37 °C and 5% CO_2_ with crude venom of *O. bauri* in different concentrations (0.3, 1, 3, 10, 30 and 60 µg/mL) or with medium alone (control). Next, parasites were washed and incubated with HeLa cell monolayers on coverslips at 2:1 (parasite: host cell) rate of infection (2 × 10^5^ tachyzoites/well/200 µL) for 24 h at 37 °C and 5% CO_2_. Cells were washed with 0.9% NaCl to remove non-adherent parasites, fixed in 10% buffered formalin for 2 h and stained with 1% toluidine blue (Sigma-Aldrich) for 5 s. Coverslips were mounted on glass slides and cells were examined under a light microscope with regards to *T. gondii* infection index (percentage of infected cells per 100 examined cells) and parasite intracellular replication (mean number of parasites per cell in 100 infected cells).

Results were expressed as percentages of inhibition of infection as well as of parasite intracellular replication for each treatment in relation to controls. The median inhibitory concentration (IC50) of venom was calculated by extrapolation of the corresponding dose-curve response on a log linear plot employing the portions of the curve that transected the 50% response point [53].

### 4.6. Statistical Analysis

Statistical analysis was carried out using the GraphPad Prism 6.0 software (1992-2012, Graphpad Sofware Inc., San Diego, CA, USA). The azocasein proteolytic activity data were analyzed by one-way ANOVA and Bonferroni multiple comparison post-test. The hemolytic activity data were analyzed by one-way ANOVA and Dunett post-test. The anticoagulant activity data were analyzed by the Student’s *t*-test. Values of *p* < 0.05 were considered statistically significant.

## 5. Conclusions

In conclusion, the present investigation describes biological and enzymatic characterization of the crude venom of *O. bauri*. The properties of the venom here reported indicate that it possesses enzymes belonging to α-fibrinogenase and demonstrates multifunctional activities, such as hemolytic, coagulant, defibrinating, antimicrobial and antiparasitic activities. This study may open interesting new structure–activity relationship perspectives for enzymes purified of the *O. bauri* crude venom with pharmacological interest for future studies related to infectious diseases.
